# Nephroprotective Effect of Methanolic Extract of *Micromeria frivaldszkyana* (Degen) Velen Against Acetaminophen Overdose in Rats

**DOI:** 10.3390/ijms27031547

**Published:** 2026-02-04

**Authors:** Elisaveta Apostolova, Vesela Kokova, Ivica Dimov, Mariya Choneva, Delyan Delev, Ilia Kostadinov, Ilia Bivolarski, Maria Koleva, Tsvetelina Mladenova, Krasimir Todorov, Anelia Bivolarska

**Affiliations:** 1 Department of Pharmacology, Toxicology, and Pharmacotherapy, Faculty of Pharmacy, Medical University of Plovdiv, Vasil Aprilov Str. 15A, 4002 Plovdiv, Bulgaria; vesela.kokova@mu-plovdiv.bg; 2 Research Institute, Medical University of Plovdiv, 4002 Plovdiv, Bulgaria; iliya.kostadinov@mu-plovdiv.bg; 3 Center for Competence “PERIMED-2”, Vasil Aprilov Blvd. 15A, 4002 Plovdiv, Bulgaria; 4 Department of Medical Biochemistry, Faculty of Pharmacy, Medical University of Plovdiv, Vasil Aprilov Str. 15A, 4002 Plovdiv, Bulgaria; ivica.dimov@mu-plovdiv.bg (I.D.); mariya.choneva@mu-plovdiv.bg (M.C.); anelia.bivolarska@mu-plovdiv.bg (A.B.); 5 Department of Pharmacology and Clinical Pharmacology, Faculty of Medicine, Medical University of Plovdiv, Vasil Aprilov Str. 15A, 4002 Plovdiv, Bulgaria; delyan.delev@mu-plovdiv.bg; 6 Department of General and Clinical Pathology, Faculty of Medicine, Medical University of Plovdiv, 4000 Plovdiv, Bulgaria; iliya.bivolarski@mu-plovdiv.bg (I.B.); mariya.koleva@mu-plovdiv.bg (M.K.); 7 Department of Botany and Biological Education, Faculty of Biology, University of Plovdiv “Paisii Hilendarski”, 24 Tsar Assen Str., 4000 Plovdiv, Bulgaria; tsvetelina.mladenova@mu-plovdiv.bg (T.M.); krasimir.todorov@mu-plovdiv.bg (K.T.); 8 Department of Bioorganic Chemistry, Faculty of Pharmacy, Medical University of Plovdiv, Vasil Aprilov Str. 15A, 4002 Plovdiv, Bulgaria

**Keywords:** paracetamol-induced kidney toxicity, biochemical markers, antioxidant activity, malondialdehide, glutathione, silymarin, rutin, rosmarinic acid, oxidative stress

## Abstract

Acetaminophen (APAP) overdose can induce potentially fatal nephrotoxicity. *Micromeria frivaldszkyana* (M. frivaldszkyana), an endemic plant to Bulgaria, has demonstrated significant antioxidant activity. This study represents the first evaluation of the nephroprotective effects of a methanolic extract of *M. frivaldszkyana* in an APAP-induced rat model of kidney injury. The aim of the study was to investigate the protective potential of orally administered *M. frivaldszkyana* methanolic extract against APAP-induced nephrotoxicity. Male Wistar rats received a one-week treatment with saline, *M. frivaldszkyana* extract (250, 400, or 500 mg/kg), rosmarinic acid (100 mg/kg), or silymarin (125 mg/kg). On day 7, renal injury was induced by oral administration of APAP (2000 mg/kg). Forty-eight hours later, blood and kidney samples were collected for biochemical and histological analyses. The extract at 500 mg/kg significantly reduced the elevated levels of serum urea (1.83 ± 0.24 vs. 3.49 ± 0.75, *p* < 0.05), creatinine (59.51 ± 2.30 vs. 72.27 ± 3.92, *p* < 0.05), and uric acid (477.55 ± 52.48 vs. 898.33 ± 65.30, *p* < 0.001), while restoring renal glutathione (GSH) levels (4.43 ± 0.19 vs. 2.64 ± 0.10, *p* < 0.001) and catalase activity (3802.78 ± 142.05 vs. 2485.03 ± 143.23, *p* < 0.01), compared with APAP-treated rats. Malondialdehyde levels were significantly reduced by the extract (25.19 ± 0.95 vs. 69.66 ± 4.11, *p* < 0.001), with similar effects observed across all tested doses. In conclusion, *M. frivaldszkyana* methanolic extract confers significant protection against APAP-induced nephrotoxicity, likely through antioxidant-mediated mechanisms, enhanced GSH restoration, and attenuation of lipid peroxidation, highlighting its potential as a nephroprotective agent.

## 1. Introduction

Paracetamol (N-acetyl-p-aminophenol, acetaminophen, APAP) is a commonly used over-the-counter antipyretic and analgesic drug. It is an affordable drug available in various formulations [1,2,3]. At therapeutic doses, APAP is considered generally safe. An overdose, however, could lead to life-threatening conditions in both humans and rodents [2]. In developed countries, APAP is considered one of the leading causes of drug overdose [3].

At therapeutic doses, the main biotransformation pathways of APAP are glucuronidation and sulfation, which lead to the production of non-toxic metabolites excreted in the urine [4]. A small percentage of the dose is metabolized via oxidation by cytochrome P450 enzymes—CYP1A2 and CYP2E1, which results in the production of N-acetyl-p-benzoquinone imine (NAPQI). The latter is a highly reactive molecule associated with oxidative stress induction, combined with mitochondrial dysfunction, and necrosis of the hepatic tissue, preceded by NAPQI’s covalent attachment to different liver proteins [4,5,6]. NAPQI’s detoxification involves its binding to glutathione (GSH), followed by renal excretion of the resulting complex. In case of APAP overdose, excessive NAPQI formation overwhelms the GSH capacity, leading to liver damage [5,7].

The kidneys are of critical importance for the excretion of APAP metabolites, and nephrotoxicity is a common complication of APAP overdose. The processes of reabsorption and secretion occur mainly in the proximal tubules, making them particularly vulnerable to APAP-induced injury [7]. Notably, recent studies suggest that nephrotoxicity can occur independently of hepatotoxicity [8]. The mechanism of APAP-induced kidney damage is likely multifaceted, involving multiple underlying pathways. The role of kidney cytochrome P450 enzymes, NAPQI formation, and oxidative stress is widely discussed. Due to its high reactivity, NAPQI can bind to intracellular macromolecules in renal cells, particularly in the proximal tubules, resulting in necrotic injury that may become life-threatening [7]. Other potentially involved enzymes in APAP-induced kidney damage include prostaglandin synthetase and N-deacetylase [8].

Toxicity caused by acetaminophen mainly results from the excessive production of various reactive oxygen species (ROS) [9]. The antioxidant enzymes glutathione peroxidase (GPx), superoxide dismutase (SOD), and catalase (CAT) neutralize ROS and protect the cell against oxidative stress [10]. Hence, insufficiency of these enzymes’ activity is strongly linked to oxidative stress-induced tissue damage. Furthermore, ROS promotes lipid peroxidation, which compromises cell membrane integrity [11]. Elevated levels of malondialdehyde (MDA) indicate excessive lipid peroxidation [12], while increased 8-hydroxy-2′-deoxyguanosine (8-OH-dG) reflects oxidative damage to DNA [13]. In the kidney, ROS trigger cellular injury and death, leading to glomerular damage, renal ischemia, and potentially leading to acute renal failure [2]. APAP-induced nephrotoxicity is also associated with elevated serum levels of renal function markers, including urea, creatinine (CR), and uric acid (UA) [14,15].

Recent studies highlight a growing interest in plant-derived agents with renoprotective potential. Silymarin is a well-known flavonoid complex obtained from *Silybum marianum* that has long been used in the management of liver diseases; however, studies have also documented its ability to protect against APAP-induced kidney damage [8,16]. Nephroprotective activity has similarly been documented for other phytochemicals [17,18], diverse plant extracts [19,20,21], and certain edible fungi, notably *Pleurotus ostreatus* [11].

*Micromeria frivaldszkyana* (*M. frivaldszkyana*) is a rare Bulgarian endemic species, belonging to the Lamiaceae family, which remains pharmacologically underexplored [22]. Recent investigations have characterized the phytochemical composition of a methanolic extract of the plant and have also evaluated its safety in subchronic and acute administration in rats [23,24]. Previously, we reported its pronounced anti-inflammatory activity in experimental rat paw edema [24], as well as its protective effects against APAP-induced hepatotoxicity [6]. Nikolova et al. (2017) [25] compared the antioxidant activity of methanolic extracts from *M. frivaldszkyana*, *M. juliana*, *M. dalmatica,* and *M. cristata* in vitro using the 2,2′-diphenylpicrylhydrazyl (DPPH) radical scavenging assay. The study demonstrated that *M. frivaldszkyana* exhibited the highest antioxidant potential [25]. The antibacterial activity of the extract was reported by Mladenova et al. (2021), showing suppression of *Listeria monocytogenes* (ATCC 19111) growth at a minimum inhibitory concentration of 10 mg/mL [22].

Metabolic profiling of the extract using UPLC-MS/MS analysis resulted in the identification of 192 compounds. Regarding the secondary metabolites, the highest amounts were registered for phenolic acids and flavonoids (mainly as flavonoid glycosides). Among the compounds with the greatest concentrations were linarin and its derivatives, rutin, chlorogenic acid, eupatorin, apigenin, and rosmarinic acid [23]. According to the literature, most of them exhibit notable antioxidant effects [26,27,28]. Moreover, recent publications have demonstrated the protective effects of linarin, rutin, apigenin, kaempferol, and chlorogenic acid against nephrotoxicity in in vivo and in vitro models [29,30,31,32].

Given these findings, we propose that the methanolic extract of *M. frivaldszkyana* could mitigate APAP-induced kidney injury in rats.

The present study aims to investigate the nephroprotective potential of *M. frivaldszkyana* methanolic extract against APAP-induced nephrotoxicity. The extent of organ injury was evaluated through histopathological examination and measurement of specific biochemical markers in both serum and kidney tissue.

## 2. Results

**Figure 1:**
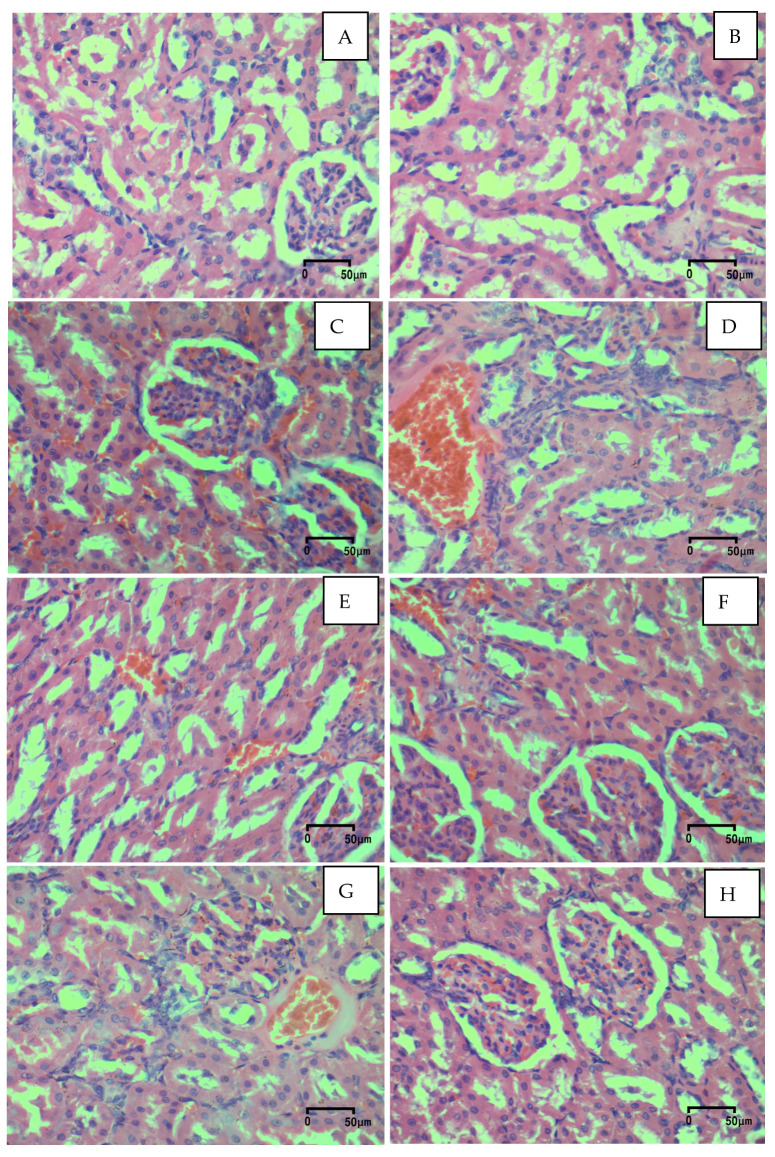
Histological examination of kidney tissue (hematoxylin and eosin staining, ×400 magnification; scale bar = 50 µm): (**A**) S (control group)—rats administered with 0.1 mL/100 g body weight (bw) saline without APAP; (**B**) ME500 group—rats administered with 500 mg/kg bw *M. frivaldszkyana* methanolic extract without APAP; (**C**) S+APAP group—rats administered with 0.1 mL/100 g bw saline and APAP; (**D**) ME250+APAP group—rats administered with 250 mg/kg bw *M. frivaldszkyana* methanolic extract and APAP; (**E**) ME400+APAP group—rats administered with 400 mg/kg bw *M. frivaldszkyana* methanolic extract and APAP; (**F**) ME500+APAP group—rats administered with 500 mg/kg bw *M. frivaldszkyana* methanolic extract and APAP; (**G**) RA+APAP group—rats administered with 100 mg/kg bw rosmarinic acid (RA) and APAP; (**H**) Sil+APAP group—rats adiminstered with 125 mg/kg bw silymarin and APAP.

As shown in Figure 1, normal renal architecture with intact glomeruli showing well-defined Bowman’s capsules and a regular arrangement of proximal and distal tubules without signs of degeneration or inflammatory infiltration was observed in both the control group (Figure 1A) and in the ME500 group (Figure 1B). In contrast, the most notable pathological alterations were observed in rats that received saline and an overdose of APAP (S+APAP) (Figure 1C). In the latter group, marked tubular degeneration, tubular luminal dilation, vascular hyperemia and hemorrhages were detected. Application of *M. frivaldszkyana* methanolic extract in the respective doses (Figure 1D–F) led to a reduction in the severity of renal derangements. These groups showed improved preservation of glomerular architecture, although the protective effect was still less pronounced than that observed in rats treated with silymarin (Figure 1H). Rats treated with RA (Figure 1G) also demonstrated a reduction in renal damage, evidenced by milder tubular degeneration, but the improvement was most notable in the rats treated with 500 mg/kg of the hydromethanolic *M. frivaldszkyana* extract and silymarin (Figure 1F,H), which exhibited the closest-to-normal histological appearance.

Quantitative histological evaluation yielded the following results:

Group 1—S (control group)—administered with 0.1 mL/100 g body weight (bw) saline; All 7 cases (100%—G0);

Group 2—ME500—administered with a water solution of the evaporated methanolic plant extract in a dose of 500 mg/kg bw; All 7 cases (100%—G0);

Group 3—S+APAP—administered with 0.1 mL/100 g bw saline; From all 7 cases—6 cases (86%—G3) and 1 case (14%—G2);

Group 4—ME250+APAP—administered with a water solution of the methanolic plant extract in a dose of 250 mg/kg bw; From all 7 cases—6 cases (86%—G1) and 1 case (14%—G0);

Group 5—ME400+APAP—administered with a water solution of the methanolic plant extract in a dose of 400 mg/kg bw; From all 7 cases—5 cases (71%—G1) and 2 cases (29%—G0);

Group 6—ME500+APAP—administered with a water solution of the hydromethanolic plant extract in a dose of 500 mg/kg bw; From all 7 cases—1 case (14%—G1) and 6 cases (86%—G0);

Group 7—RA+APAP—administered with 100 mg/kg bw RA; From all 7 cases—4 cases (57%—G1) and 3 cases (43%—G0);

Group 8—Sil+APAP—administered with 125 mg/kg bw silymarin. From all 7 cases—1 case (14%—G1) and 6 cases (86%—G0).

**Figure 2:**
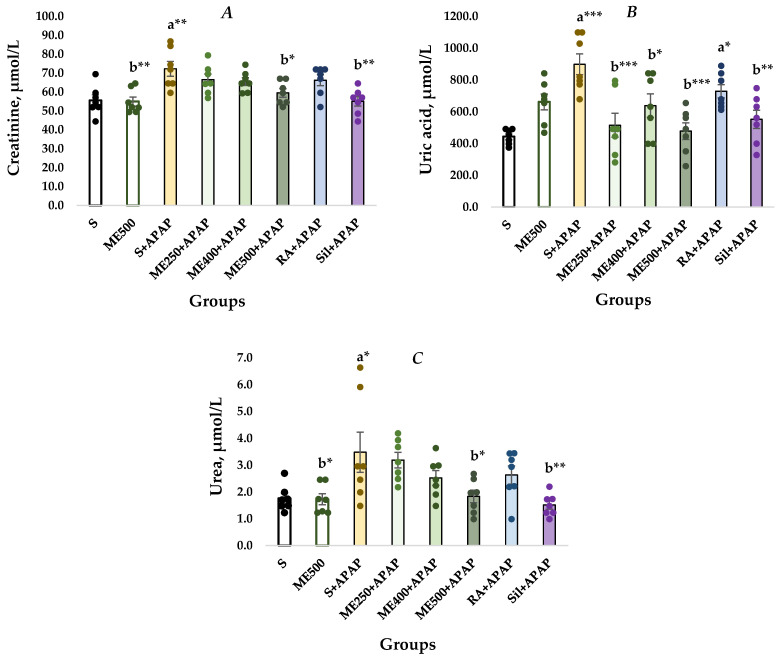
Serum levels of (**A**) creatinine; (**B**) uric acid, and (**C**) urea. Data are presented as mean ± SEM and raw values, represented by the colored circles. One-way ANOVA, followed by Tukey’s post hoc test, revealed significant differences between the groups: a*—*p* < 0.05 vs. S group; a**—*p* < 0.01 vs. S group; a***—*p* < 0.001 vs. S group; b*—*p* < 0.05 vs. S+APAP group; b**—*p* < 0.01 vs. S+APAP group; b***—*p* < 0.001 vs. S+APAP group. **S** (control group)—rats administered with 0.1 mL/100 g body weight (bw) saline without APAP; ME500 group—rats administered with 500 mg/kg bw *M. frivaldszkyana* methanolic extract without APAP; S+APAP group—rats administered with 0.1 mL/100 g bw saline and APAP; ME250+APAP group—rats administered with 250 mg/kg bw *M. frivaldszkyana* methanolic extract and APAP; ME400+APAP group—rats administered with 400 mg/kg bw *M. frivaldszkyana* methanolic extract and APAP; ME500+APAP group—rats administered with 500 mg/kg bw *M. frivaldszkyana* methanolic extract and APAP; RA+APAP group—rats administered with 100 mg/kg bw rosmarinic acid (RA) and APAP; Sil+APAP group—rats adiminstered with 125 mg/kg bw silymarin and APAP.

In consistency with the histological data, the animals administered with saline and APAP had increased levels of serum markers reflecting renal damage. Rats treated with S+APAP showed significantly higher serum creatinine levels than control rats (72.27 ± 3.92 vs. 55.54 ± 2.93, *p* < 0.01). Decreased creatinine levels were observed in groups ME500+APAP and Sil+APAP in comparison to the S+APAP group (59.51 ± 2.30 vs. 72.27 ± 3.92, *p* < 0.05; 55.08 ± 2.55 vs. 72.27 ± 3.92, *p* < 0.01) (Figure 2A).

A significantly increased uric acid concentration was detected in the S+APAP and RA+APAP rats in comparison to the S group (898.33 ± 65.30 vs. 443.23 ± 17.73, *p* < 0.001; 728.57 ± 41.94 vs. 443.23 ± 17.73, *p* < 0.05), as shown in Figure 2B. The serum UA levels in the ME250+APAP, ME400+APAP, ME500+APAP, and Sil+APAP groups were significantly lower compared to the S+APAP group (514.28 ± 75.66 vs. 898.33 ± 65.30, *p* < 0.001; 637.85 ± 73.87 vs. 898.33 ± 65.30, *p* < 0.05; 477.55 ± 52.48 vs. 898.33 ± 65.30, *p* < 0.001; 551.76 ± 56.86 vs. 898.33 ± 65.30, *p* < 0.01).

Figure 2C demonstrates significantly higher urea concentration in the S+APAP rats in comparison to the controls (S group) (3.49 ± 0.75 vs. 1.76 ± 0.18, *p* < 0.05). The urea levels were significantly lower in the ME500+APAP and Sil+APAP groups in comparison to S+APAP (1.83 ± 0.24 vs. 3.49 ± 0.75, *p* < 0.05; 1.51 ± 0.15 vs. 3.49 ± 0.75, *p* < 0.05).

**Figure 3:**
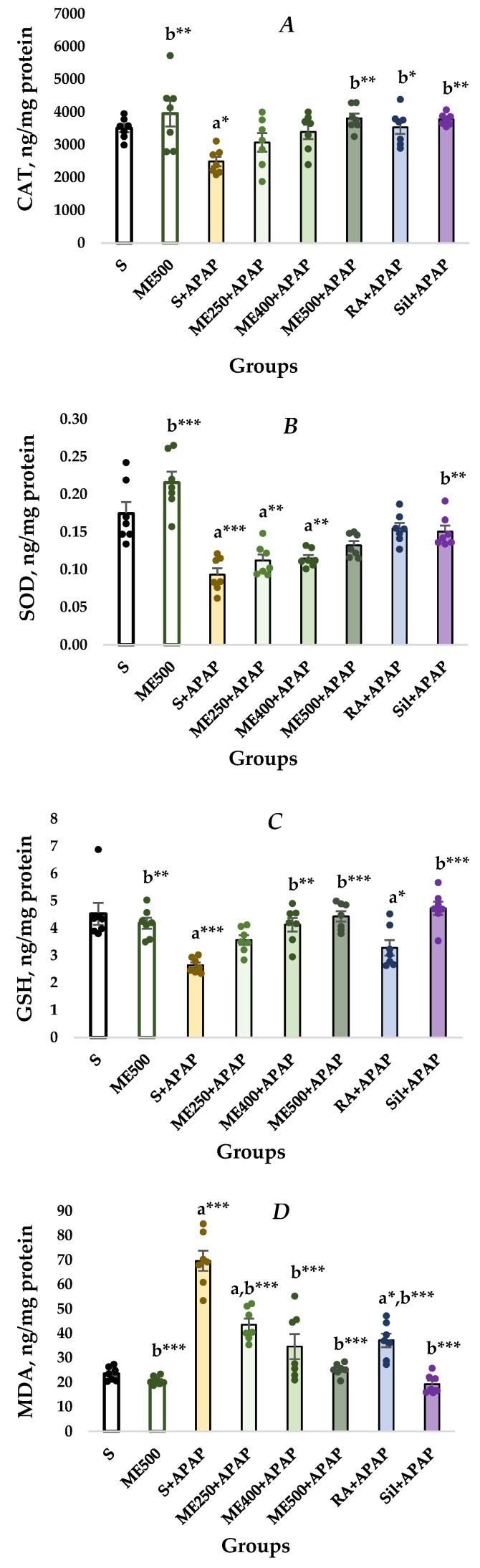

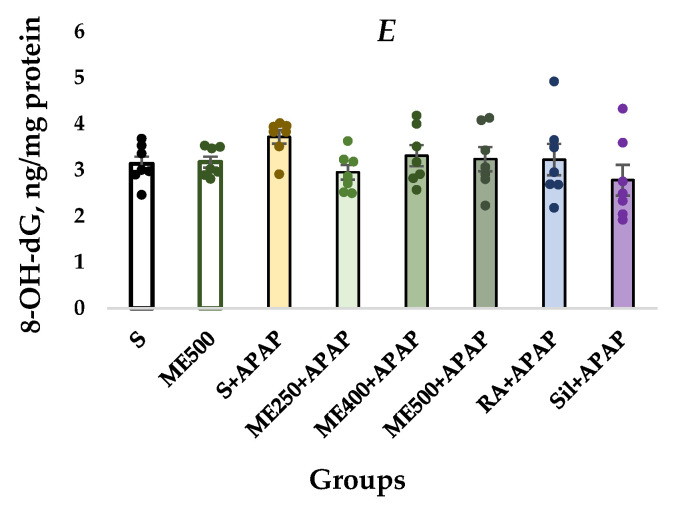
Kidney biochemical markers. (**A**) catalase; (**B**) superoxide dismutase; (**C**) reduced glutathione; (**D**) malondialdehyde; (**E**) 8-hydroxy-deoxyguanosine. Data are presented as mean ± SEM and raw values, represented by the colored circles. One-way ANOVA, followed by Tukey’s post hoc test, revealed significant differences between the groups: a*—*p* < 0.05 vs. S group; a**—*p* ≤ 0.01 vs. S group; a***—*p* < 0.001 vs. S group; b*—*p* < 0.05 vs. S+APAP group; b**—*p* < 0.01 vs. S+APAP group; b***—*p* < 0.001 vs. S+APAP group. S (control group)—rats administered with 0.1 mL/100 g body weight (bw) saline without APAP; ME500 group—rats administered with 500 mg/kg bw *M. frivaldszkyana* methanolic extract without APAP; S+APAP group—rats administered with 0.1 mL/100 g bw saline and APAP; ME250+APAP group—rats administered with 250 mg/kg bw *M. frivaldszkyana* methanolic extract and APAP; ME400+APAP group—rats administered with 400 mg/kg bw *M. frivaldszkyana* methanolic extract and APAP; ME500+APAP group—rats administered with 500 mg/kg bw *M. frivaldszkyana* methanolic extract and APAP; RA+APAP group—rats administered with 100 mg/kg bw rosmarinic acid (RA) and APAP; Sil+APAP group—rats adiminstered with 125 mg/kg bw silymarin and APAP.

The results from the histological examination are further confirmed by the results on the biochemical tissue markers assessing the oxidative stress and antioxidant defense status (Figure 3). As shown in Figure 3A, renal CAT levels were significantly decreased in the S+APAP group compared to the controls (2485.03 ± 143.23 vs. 3501,37 ± 121,25, *p* < 0.05) and significantly increased in the ME500, ME500+APAP, RA+APAP and Sil+APAP group, in comparison to group S+APAP (3947.18 ± 396.67 vs. 2485.03 ± 143.23, *p* < 0.01; 3802.78 ± 142.05 vs. 2485.03 ± 143.23, *p* < 0.01; 3527.12 ± 199.54 vs. 2485.03 ± 143.23, *p* < 0.05; 3771.83 ± 67.45 vs. 2485.03 ± 143.23, *p* < 0.01).

As shown in Figure 3B, the levels of SOD in the S+APAP, ME250+APAP and ME400+APAP groups were significantly lower compared to the S group (0.09 ± 0.009 vs. 0.17 ± 0.02, *p* < 0.001; 0.11 ± 0.008 vs. 0.17 ± 0.02, *p* < 0.01; 0.11 ± 0.004 vs. 0.17 ± 0.02, *p* < 0.01). Groups ME500 and Sil+APAP demonstrate a significant increase in SOD levels compared to the S+APAP group (0.22 ± 0.01 vs. 0.09 ± 0.009, *p* < 0.001; 0.15 ± 0.008 vs. 0.09 ± 0.009, *p* < 0.01).

A significant decrease in GSH levels was observed in the S+APAP and RA+APAP groups compared to the control rats (2.64 ± 0.10 vs. 4.53 ± 0.40, *p* < 0.001; 3.28 ± 0.28 vs. 4.53 ± 0.40, *p* < 0.05). Additionally, GSH levels were significantly elevated in ME500, ME400+APAP, ME500+APAP and Sil+APAP groups in comparison to S+APAP group (4.18 ± 0.20 vs. 2.64 ± 0.10, *p* ≤ 0.001; 4.12 ± 0.25 vs. 2.64 ± 0.10, *p* < 0.01; 4.43 ± 0.19 vs. 2.64 ± 0.10, *p* < 0.001; 4.73 ± 0.24 vs. 2.64 ± 0.10, *p* < 0.001), as shown in Figure 3C.

The conducted analysis revealed significantly increased MDA renal concentration in the S+APAP, ME250+APAP, and RA+APAP groups in comparison to the controls (69.66 ± 4.11 vs. 23.47 ± 1.07, *p* < 0.001; 43.54 ± 2.51 vs. 23.47 ± 1.07, *p* < 0.001; 37.16 ± 2.78 vs. 23.47 ± 1.07, *p* < 0.05). The obtained results (Figure 3D) indicate significantly decreased MDA levels in the ME500, ME250+APAP, ME400+APAP, ME500+APAP, RA+APAP, and Sil+APAP groups compared to the S+APAP group (20.75 ± 0.64 vs. 69.66 ± 4.11, *p* < 0.001; 43.54 ± 2.51 vs. 69.66 ± 4.11, *p* < 0.001; 34.63 ± 5.11 vs. 69.66 ± 4.11, *p* < 0.001; 25.19 ± 0.95 vs. 69.66 ± 4.11, *p* < 0.001; 37.16 ± 2.78 vs. 69.66 ± 4.11, *p* < 0.001; 19.26 ± 1.46 vs. 69.66 ± 4.11, *p* < 0.001).

The statistical analysis revealed no significant differences in the 8-OH-dG levels among the groups (Figure 3E).

## 3. Discussion

APAP overdose is usually associated with liver injury; however, renal toxicity should not be underestimated, as it can also result in a fatal outcome. As mentioned above, the toxicity of this compound is probably due to increased NAPQI production [7]. CYP450 enzymes are essential for its generation, while binding to GSH is the main route for its detoxification. In cases of APAP overdose, excessive NAPQI formation occurs alongside a reduction in GSH levels in both the liver and kidneys. NAPQI induces its toxicity by covalent binding to cellular proteins [7]. However, the mechanism of APAP-induced kidney damage has still not been fully elucidated, and involvement of specific enzymatic activities cannot be excluded [8].

Biomarkers commonly used in clinical practice to assess renal function include urea, CR, and UA. Impaired renal function leads to decreased clearance of these molecules and a subsequent increase in their serum levels [7,21]. The present study revealed a significant elevation in the urea, CR, and UA levels in rats with APAP overdose, indicating kidney dysfunction. Pre-treatment with the methanolic *M. frivaldszkyana* extract and silymarin decreased the damage severity. The methanolic extract decreased the elevated levels in a dose-dependent manner; however, statistical significance was reached only at the 500 mg/kg dose of the plant extract. These results are consistent with those reported by other authors, whose studies detected increased levels of urea, CR, and UA after a single oral APAP overdose and amelioration following silymarin pre-treatment [7,8,21]. The histopathological observation (Figure 1) further supported these findings.

To our knowledge, there are no published reports on the effect of RA in APAP-induced nephrotoxicity. In other models, however, RA demonstrated nephroprotective activity. Domitrović et al. (2014) [33] reported reduced CR levels after RA treatment in cisplatin-induced nephrotoxicity, whereas our study did not detect significant CR changes. These discrepancies can be explained by the different nephrotoxic agents (APAP vs. cisplatin) and animal species (rats vs. mice) used in the reports [33]. Tavafi et al. (2011) explored the protective effect of RA in a model of gentamicin-induced nephrotoxicity in Sprague Dawley rats and found a significant decrease in the levels of urea and CR in rats with RA application [34]. Variability in rat strain, experimental protocol, and toxicant used may account for these differences.

APAP-induced nephrotoxicity is manifested by alterations in urine volume, GSH and CR levels, accumulation of lipid peroxidation products, and decreased glomerular filtration [19]. The involvement of oxidative stress in APAP overdose-induced kidney toxicity was described above and is further underlined by the detected low CAT and SOD activities observed in the S+APAP group. Recent research by Olukanni et al. (2025) and Reshi et al. (2020) demonstrated elevated levels of both antioxidant enzymes after silymarin pre-treatment [7,21]. No reports on the effect of RA on these enzymes in APAP-induced nephrotoxicity were found in the scientific literature. However, in a model of gentamicin-induced nephrotoxicity, pre-treatment with RA (100 mg/kg for 14 days) elevated the levels of CAT and SOD in a kidney homogenate, and this result aligns with the current findings [34]. Xiang et al. (2022) reported a significant increase in antioxidant enzymes, including SOD and CAT, in a mouse model of cisplatin-induced kidney injury following RA treatment [35]. The divergent outcomes in these two experiments can be attributed to the different nephrotoxic agents, animal species, and administration routes. In the current study, a dose-dependent increase in both CAT and SOD was observed after *M. frivaldszkyana* extract treatment, although statistical significance was reached only for CAT at the highest dose (Figure 3A). This observation suggests that the upregulation of the activities of the assessed antioxidant enzymes is one of the mechanisms underlying the detected nephroprotective effect exerted by the plant extract. However, it is also possible that the major role is played by other mechanisms. This hypothesis is supported by our previous work, which reported no significant changes in hepatic CAT and SOD after treatment with the extract of rats with APAP overdose [6].

Another important molecule, engaged in the protection of kidney tissue against NAPQI-mediated damage, is GSH. As expected, APAP markedly reduced its levels, while silymarin induced an increase, and these findings align with Reshi et al. (2020) [7]. In contrast, Olukanni (2025) did not register changes in renal GSH after APAP or silymarin application [21]. It is highly possible that the reason for the different results is the number of animals in the groups. The present study was performed on groups of 8 animals, the study of Reshi et al. (2020) was carried out on groups of 6 animals, while the study of Olukanni was performed on groups of 5 animals [7]. Larger sample sizes increase the likelihood of detecting significant effects (personal communication). Another possible explanation lies in the different experimental protocols employed in both studies. The current study demonstrated that exposure of the animals to RA did not alter the renal GSH levels. In contrast, Xiang et al. (2022) and Tavafi et al. (2011) reported a significant increase in this biomarker, and the reasons are discussed in the previous paragraph [34,35]. The methanolic extract of *M. frivaldszkyana* elicited a dose-dependent elevation of the kidney GSH with doses of 400 mg/kg and 500 mg/kg reaching statistical significance compared to APAP alone. These findings suggest that restoration of GSH may be a critical mechanism underlying the extract’s nephroprotective activity.

The levels of MDA, another oxidative stress-related biomarker, were significantly decreased in the kidneys of rats treated with silymarin and RA. Tavafi et al. 2011 reported similar results for RA in a model of nephrotoxicity induced by gentamicin [34]. The methanolic extract of *M. frivaldszkyana* significantly decreased the MDA levels dose-dependently, with the highest dose restoring the values close to control levels. This suggests reduced lipid peroxidation after treatment with *M. frivaldszkyana* extract. Previously, a similar impact of this extract was reported for the liver MDA levels in the same model of APAP toxicity [6]. Overall, the data obtained in the current study suggests that the *M. Frivaldszkyana* methanolic extract possesses a protective effect against APAP overdose–induced oxidative stress in kidney tissue.

The antioxidant potential of plants from the Lamiaceae family has been widely documented [22,36]. A comparative in vitro study on four *Micromeria* species (*M. Juliana*, *M. Frivaldszkyana*, *M. dalmatica*, and *M. cristata*) was performed with a DPPH assay [25]. The highest antioxidant activity was registered for the *M. frivaldszkyana* and *M. dalmatica* methanolic extracts [25]. This result is in accordance with more recent reports [22,37]. The latter study reported that *M. Frivaldszkyana*’s antioxidant effect was significantly higher compared to a variety of Bulgarian medicinal plants as assessed in the oxygen radical absorbance capacity (ORAC) assay (3250.5 ± 208.1 μmol TE/g) [37].

In many cases, the biological activity of plant extracts can be attributed to their main compounds. Acacetin 7-O-rutinoside (also known as linarin) is a flavonol glycoside, which was identified in plant species from the Lamiaceae and Asteraceae families, and the *Micromeria*, *Mentha* and *Satureja* genera have been reported to contain the highest amounts of linarin [38]. Many biological activities, such as antioxidant, anti-inflammatory, and hepatoprotective properties, have been reported for this compound [38,39]. Linarin was found to decrease elevated serum UA and CR levels in a model of hyperuricemia in mice. The same study demonstrated increased CAT, SOD, and GSH levels in vitro and in linarin-treated mice [40]. Additionally, serum MDA levels were decreased, supporting the hypothesis of its antioxidant and renoprotective effect [40]. Similar results (increased CAT, SOD, and GSH; decreased MDA and CR) were reported by Qi et al. (2024) in a model of cisplatin-induced nephrotoxicity in rats [29]. When taken orally, some bioactive compounds may be subjected to degradation in the gastrointestinal tract or decreased absorption through the mucus, resulting in decreased plasma levels [41]. However, Li et al. (2019) published a pharmacokinetic study revealing rapid absorption of linarin following oral administration in rats [42]. Based on this, we hypothesize that the observed antioxidant effect may be partially related to the high linarin content in the extract. However, it is more likely that the registered effect of the extract is related to all present compounds, not only to those with the highest biological activity [43].

Nephroprotective properties have also been described for rutin, chlorogenic acid, apigenin, naringin, and rosmarinic acid in various models of drug-induced kidney toxicity. However, only one study has reported such activity in APAP-induced nephrotoxicity. Adil et al. (2016) [44] found a significant increase in the serum CR level, while oral treatment with naringin reduced these levels. Similar results were reported for MDA in kidney tissue, while the levels of SOD and GSH were increased. However, to be absorbed, naringin needs to undergo transformation to naringenin [44]. Naringin was not identified in the methanolic extract of *M. frivaldszkyana*, but low concentrations of naringenin were registered [23]. Additionally, naringenin was reported to ameliorate the nephrotoxicity induced by gentamicin, and the results were attributed to its antioxidant and anti-inflammatory properties [45].

The second most abundant compound in the *M. frivaldszkyana* extract is chlorogenic acid (5-caffeoylquinic acid) [23]. This phenolic compound demonstrated antioxidant, anti-inflammatory, anti-apoptotic, autophagy suppressive, and free radical scavenging properties in models of vancomycin-induced nephrotoxicity in rats [32] and cisplatin-induced kidney damage in mice [46].

Rutin (quercetin-3-O-rutinoside) is a flavonoid with antioxidant, anti-inflammatory, and anti-apoptotic effects and was found effective against drug-induced kidney damage [30]. Rutin ameliorated the increased serum CR levels and restored the levels of SOD, CAT, GSH, and MDA in renal tissue in a rat model of nephrotoxicity induced by vancomycin [47] and gentamicin [48]. Similar changes in CR, GSH, and MDA levels were reported for cisplatin-induced toxicity [49]. Based on these reports and the high concentration of rutin in the methanolic extract (the third most abundant compound), we propose that rutin has a significant role in the registered nephroprotective and antioxidant effects.

Hussein et al. (2022) [50] reported decreased levels of urea and CR post administration of apigenin in a model of gentamicin-induced nephrotoxicity in rats. Increased levels of SOD, CAT, and GSH were also observed in the treated animals in comparison to the rats treated with gentamicin alone [50]. Another study reported restoration of SOD, MDA, and GSH levels after apigenin application in mice with doxorubicin-induced nephrotoxicity [51].

Rosmarinic acid is an ester of 3, 4-dihydroxyphenyl lactic acid and caffeic acid. This phenolic compound is abundant in the Lamiaceae family and demonstrated cardioprotective, hepatoprotective, nephroprotective, antibacterial, antioxidant, and anti-inflammatory properties in vivo and in vitro [52]. Among all hydroxycinnamic acid derivatives, RA is considered to have among the strongest antioxidant activity. It has been shown that RA decreases the levels of urea and CR in gentamicin-induced nephrotoxicity in rats [34,52].

Various authors have proven antioxidant effects for the main components of the studied extract by modulating specific cellular signaling pathways. Polyphenols in *M. frivaldszkyana* (rutin, naringenin, apigenin, linarin, chlorogenic acid, and rosmarinic acid) exert antioxidant and anti-inflammatory effects via activation of the nuclear factor erythroid 2-related factor 2 (Nrf2) pathway leading to upregulation of cytoprotective antioxidant enzymes such heme oxygenase-1 (HO 1), NAD(P)H:quinone-oxidoreductase-1 (NQO1), and glutamate–cysteine ligase catalytic subunit (GCLC) and suppression of NF-κB signaling (TNF-α, IL-6, IL-1β), following the principle of hormesis: moderate doses trigger adaptive, stress-resistant responses, whereas high doses are toxic [53,54,55,56]. Linarin, rutin, apigenin, RA, chlorogenic acid, and naringenin exhibit direct ROS scavenging and indirect (Nrf2-mediated) antioxidant effects. They activate the Nrf2 pathway, HO-1, NQO1, and GCLC, thereby enhancing endogenous cellular defenses [40,57,58,59,60,61]. The antioxidant activity leads to suppression of ROS-sensitive inflammatory and stress pathways, including the nuclear factor-kappa B (NF-κB) pathway, whose activation by ROS triggers the expression of pro-inflammatory cytokines (TNF-α, IL-6, IL-1β). All six compounds inhibit NF-κB nuclear translocation [40,62,63,64,65,66] and downstream pro-inflammatory gene transcription. Beyond the NF-κB pathway, RA, linarin, rutin, apigenin, chlorogenic acid, and naringenin suppress ROS-mediated MAPK activation (JNK, p38, ERK), preventing apoptosis or inflammation [39,67,68,69,70,71]. Additionally, these compounds inhibit apoptosis or protect cells by reducing pro-apoptotic signaling in oxidative-stress or injury models. They increase the level of anti-apoptotic protein Bcl-2 and inhibit the up-regulation of Bax expression and caspase-3, stabilizing the Bcl-2/Bax ratio [72,73,74,75,76,77]. Collectively, these findings demonstrate that the antioxidant effects of the main extract’s components are mechanistically integrated with Nrf2 pathway activation, suppression of ROS-activated NF κB and MAPK pathways, and inhibition of apoptosis. We propose that these mechanisms may at least partially explain the established nephroprotective effect of the methanolic extract of *M. frivaldszkyana*.

Our dose-dependent reduction in serum uric acid, together with increased CAT and GSH levels and decreased MDA following administration of 500 mg/kg ME500, supports hormetic activation of endogenous antioxidant defenses that counteract the pro-oxidant effects of APAP in a dose-dependent manner. The optimal dose (500 mg/kg) appears to induce Nrf2 and other stress-adaptive genes, whereas excessive doses may promote lipid peroxidation, a hallmark of hormetic responses [55,78,79]. Taken together, these findings suggest that the observed nephroprotective effect could be attributed to the high antioxidant activity, increased GSH, and decreased lipid peroxidation. Additionally, the modulation of antioxidant, inflammatory, and apoptotic pathways may explain the established effect. Given its potent antioxidant properties, further studies are warranted to explore the efficacy of this extract in other models of toxicity and in different organs.

The present study has several limitations that should be acknowledged. First, the assessment of APAP-induced nephrotoxicity was limited to male Wistar rats; therefore, the findings may not be valid for female animals, other rat strains and species, or alternative routes of APAP administration. Second, the extract was administered for seven days prior to APAP exposure, and different pre-treatment durations or post-treatment regimens may influence the observed outcomes. Third, nephrotoxicity was induced by a single acute APAP dose; thus, the results may differ under conditions of repeated or chronic exposure. Finally, the study primarily focused on biochemical and histological endpoints, and no molecular signaling pathways were investigated. Consequently, the precise intracellular mechanisms underlying the observed nephroprotective effects remain to be elucidated in future studies.

## 4. Materials and Methods

The experiments in the present study were approved by the Ethics Committee of the Medical University of Plovdiv, Bulgaria (protocol number: 6/5 October 2023) and the Bulgarian Food Safety Agency (permit number: 352/30 May 2023), and comply with the EU Directive (2010/63/EU) and the ARRIVE guidelines for working with experimental animals. A permit from the Ministry of Environment and Water (996/9 August 2023) was obtained for the collection of the plant material.

### 4.1. Chemicals and Reagents

Paracetamol (≥98.0%, batch No. MKCS3304), rosmarinic acid (≥96.0%, batch No. BCCJ6033), silymarin (≥30.0%, batch No. BCCH4151), and methanol (≥99.8%, cat. No. 179337) were sourced from Merck SA, Germany. Hematoxylin G3 (cat. № 294/HEMG3-OT-2.5L), eosin Y (1% aqueous solution, cat. № 294/EOY-10-OT-2.5L), Histanol 95 (cat. № 294/H95-5L), Histanol 100 (cat. № 294/H100-5L), formaldehyde 4% (10% neutral buffered formalin, cat. № 294/FNB4-10L), xylol (cat. № 348/3410/20), and acetone (cat. № 48/3413/5) were obtained from BIOCARE Medical, Pacheco, CA, USA.

### 4.2. Plant Material and Preparation of the Methanolic Extract

Approximately 800 g of *M. frivaldszkyana* aerial parts (fresh biomass) were collected from Bulgarka Nature Park, a floristic region of Middle Stara Planina, during 2023–2024 as previously reported [23,24]. The specimen number (062648) was obtained upon deposition at the herbarium of the Agricultural University in Plovdiv. The collected plant material was subjected to air-drying in a shaded area at 22 ± 2 °C until the leaf stalks broke when bent and the petioles fell apart when pressure was applied. The dried material was afterwards milled on a laboratory mill (GRINDOMIX GM200, RETSCH GmbH, Haan, Germany) to a powder with particles of approximately 400 μm in size.

The methanolic extract was prepared as follows: 10 g of the plant powder was soaked in 70% (*v*/*v*) methanol (1:10 *w*/*v*) in a beaker wrapped in aluminum foil at room temperature for 24 h. The macerate was continuously stirred, to improve extraction efficiency, the sample was subjected to triple ultrasonication for 15 min at 30 °C. The sample was afterwards centrifuged for 15 min at 6000 rpm, and the obtained supernatant underwent filtration with filter paper Whatman No. 1 (Sigma-Aldrich, Burlington, MA, USA). All listed steps were repeated two more times on the residual plant material, and the three obtained extracts were combined. Subsequent solvent removal was conducted on a rotary evaporator (Heidolph, Schwabach, Germany) under reduced pressure at 50 °C. The total extraction yield was 5.48 g of dry extract or 54.8% of the initial dry plant mass equivalent [6,23].

### 4.3. Animals and Treatment

The subjects of the experiment were 56 male Wistar rats with an average weight of 235 ± 25 g. The animals were randomized into 8 groups (*n* = 7). Each group was housed in a separate plastic cage and maintained under controlled laboratory conditions (22 ± 1 °C, 45% relative humidity, 12:12 h light/dark cycle) with unrestricted access to standard rat chow and water.

Ahmed et al. (2023) described an experimental protocol for APAP-induced liver toxicity [5], and Reshi et al. (2020) demonstrated that the same single dose (2 g/kg, per os) also induced nephrotoxicity [7]. Following a one-week acclimatization, the rats were administered oral treatments for 7 days in accordance with group designations as follows:

1. Group 1—S (control group)—administered with 0.1 mL/100 g body weight (bw) saline;
2. Group 2—ME500—administered with a water solution of the evaporated hydromethanolic plant extract in a dose of 500 mg/kg bw;
3. Group 3—S+APAP—administered with 0.1 mL/100 g bw saline;
4. Group 4—ME250+APAP—administered with a water solution of the hydromethanolic plant extract in a dose of 250 mg/kg bw;
5. Group 5—ME400+APAP—administered with a water solution of the hydromethanolic plant extract in a dose of 400 mg/kg bw;
6. Group 6—ME500+APAP—administered with a water solution of the hydromethanolic plant extract in a dose of 500 mg/kg bw;
7. Group 7—RA+APAP—administered with 100 mg/kg bw RA;
8. Group 8—Sil+APAP—administered with 125 mg/kg bw silymarin.

After six days of the treatment described above, the rats were subjected to a 12 h fast with unlimited water access. On the seventh day, groups 3, 4, 5, 6, 7, and 8 were administered 2000 mg/kg APAP using a gastric tube. After 3 h, each group received the respective extract or compound treatment. The rats were euthanised 48 h post APAP gavage. Whole blood samples were immediately collected from the animals, and the serum was obtained according to a standard protocol [centrifugation in an MPW-352R centrifuge (MPW Med. Instruments, Warsaw, Poland) at 3000 rpm for 10 min at 4 °C] and was immediately frozen at −80 °C until the biochemical analysis. The kidneys were excised and divided into parts, with one portion used for the histopathological analysis and stored at −80 °C. The frozen kidneys were afterwards used for the preparation of a tissue homogenate that was subjected to biochemical analysis.

The selected duration of treatment preceding the APAP administration was based on a preliminary literature review and other studies that assessed different natural compounds and/or plant extracts and their nephroprotective effects in models of APAP-induced kidney toxicity [80,81]. The doses of the methanolic extract were selected as specified by Hanafy et al. (2016), who recommended doses of 1/10 and 1/20 of the assessed LD_50_ [82]. Our previous research determined that a single administration of up to 5000 mg/kg plant extract did not exert toxic effects [4]. Apart from the doses corresponding to 1/20 and 1/10 of LD_50_—250 and 500 mg/kg, respectively, an intermediate dose of 400 mg/kg was added to the protocol for a better characterization of the dose-dependent effects of the extract.

The used RA dose was adapted to the research of Hasanein et al. (2017) [12], which reported the compound’s hepatoprotective effects in a model of APAP-induced liver damage. Additionally, a study by Fadlalla (2020) demonstrated the nephroprotective effect of rosemary extract in a dose of 125 mg/kg bw [83]. The dose for the positive control silymarin, known for its hepatoprotective activity, was also selected in accordance with published data [7,8,80,84].

### 4.4. Histopathological Assessment and Kidney Injury Biomarker Evaluation

#### 4.4.1. Histopathological Observation

The applied study protocol was reported previously [24] and conformed to the research of other authors [85,86,87]. The preparation of the kidney histological samples was conducted in three steps:

1. Tissue immersion in 10% neutral-buffered formalin for 24 h immediately after excision, and further rinsing and soaking in distilled water for 30 min to eliminate the residual fixative.
2. Dehydration of the tissues via progressive consecutive immersion in 95% and 100% ethanol (Histanol^®^ 100%) for 4–5 h each.
3. Treatment with acetone for 20 min and then xylol for 30 min, embedding in molten paraffin and block formation, preparation of 5 μm thick slices, staining with hematoxylin and eosin, and microscopic examination.

Two independent pathologists without knowledge of the randomization of the animals into groups performed the histopathological analysis and observed the samples on Zeiss AXIO Scope A1 (Carl Zeiss Microscopy GmbH, Jena, Germany) and Leica DM500 (Leica Microsystems GmbH, Wetzlar, Germany) microscopes. The renal tissue was evaluated for histological evidence of changes in the normal architecture, necrosis, apoptosis, inflammation, and vascular alterations.

All renal specimens obtained from euthanized rats were examined by light microscopy and graded into five categories using a 0–5 scoring system, as described and adapted by Ahmed et al. [88].

Grade 0—normal histology, characterized by preserved renal architecture, intact proximal and distal tubules, and clearly delineated vascular and urinary poles, with the presence of a few mitotic cells.

Grade 1—tubular epithelial cell degeneration without significant features of necrosis or apoptosis.

Grades 2–5—<25%, <50%, <75%, and >75% of tubules, respectively, showing tubular epithelial cell necrosis and/or apoptosis, accompanied by other concomitant histopathological alterations.

#### 4.4.2. Preparation of Kidney Homogenates and Evaluation of Tissue-Specific Toxicity Markers

Kidney homogenates were prepared using a Polytron mechanical homogenizer (KINEMATICA, Malters, Switzerland). The frozen tissue was brought to ambient temperature, and after defrosting, the samples were rinsed and homogenized in ice-cold lysis buffer (0.1 M PBS, pH 7.4), to which Triton X-100 was added (1:9 *w/v*) [89]. The procedure was followed by centrifugation (10,000 rpm, 10 min at 4 °C) on an MPW-352R centrifuge (Warsaw, Poland) [90]. The supernatant was used for the determination of the tissue levels of: CAT, SOD1, GSH, MDA, and 8-OH-dG for evaluation of the redox homeostasis. The analysis of the listed markers was performed using an enzyme-linked immunosorbent assay (ELISA) method on an ELISA microplate reader (HumanReader, HUMAN, Wiesbaden, Germany). The following ELISA kits were purchased from Elabscience Biotechnology Inc. (Houston, TX, USA): SOD1 (Superoxide Dismutase 1, Soluble), GSH (Glutathione), MDA (Malondialdehyde), 8-OHdG (8-Hydroxydeoxyguanosine), while the ELISA Kit for Catalase (CAT) was purchased from Cloud-Clone Corp. (Katy, TX, USA).

#### 4.4.3. Biochemical Markers in Serum

The obtained serum was subjected to spectrophotometric evaluation of U, CRE, and UA levels on an Evolution 300 UV-Vis spectrophotometer (Thermo Fisher Scientific, Waltham, MA, USA). Kits were purchased from Human GmbH (Weisbaden, Germany), and the included instructions were followed during the conduction of the analysis.

### 4.5. Statistical Analysis

The statistical software SPSS (17.0, IBM, New York, NY, USA) was used for statistical analysis. One-way analysis of variance (ANOVA) followed by Tukey’s post hoc test was used for intergroup comparisons. Data are presented as mean ± SEM values. Statistical significance was set at *p* ≤ 0.05.

## 5. Conclusions

The present study provides the first experimental evidence for the nephroprotective effects of a methanolic extract of *Micromeria frivaldszkyana* in an APAP-induced rat model of kidney injury. A seven-day pre-treatment with the extract significantly attenuated APAP-induced renal dysfunction and oxidative stress. The observed protective effects are likely associated with enhanced antioxidant status, increased glutathione levels, and reduced lipid peroxidation. The major phytochemical constituents identified in the extract, such as rutin, naringenin, apigenin, linarin, chlorogenic acid, and rosmarinic acid, may contribute to these effects; however, the precise molecular mechanisms involved require further investigation.

## Data Availability

The raw data supporting the conclusions of this article will be made available by the authors on request.

## Abbreviations

The following abbreviations are used in this manuscript:

8-OH-dG	8-hydroxy-2′-deoxyguanosine
APAP	Acetaminophen (paracetamol)
CAT	Catalase
CR	Creatinine
CYP	Cytochrome P450
DNA	Deoxyribonucleic acid
DPPH	2,2′-diphenylpicrylhydrazyl
ELISA	Enzyme-linked immunosorbent assay
GCLC	Glutamate–cysteine ligase catalytic subunit
GSH	Glutathione
HO-1	Heme oxygenase-1
MDA	Malondialdehyde
NAC	N-acetylcysteine
NAPQI	N-acetyl-p-benzoquinone imine
NF-κB	Nuclear factor-kappa B
Nrf2	Nuclear factor erythroid 2-related factor 2
NQO1	NAD(P)H:quinone-oxidoreductase-1
ORAC	Oxygen radical absorbance capacity
RA	Rosmarinic acid
ROS	Reactive oxygen species
SOD	Superoxide dismutase
UA	Uric acid
UPLC-MS/MS	Ultra-performance liquid chromatography tandem mass spectrometry
